# Airway Management in Pregnancy: A Case of Successful Treatment of Subglottic Stenosis

**DOI:** 10.7759/cureus.92290

**Published:** 2025-09-14

**Authors:** Linh Ton, Ritika Modi, Diane Choi, Gisele J Wakim

**Affiliations:** 1 Anesthesiology, University of Miami Miller School of Medicine, Miami, USA; 2 Anesthesiology, University of Miami Miller School of Medicine, Jackson Memorial Hospital, Miami, USA; 3 Clinical Anesthesiology, University of Miami Miller School of Medicine, Jackson Memorial Hospital, Miami, USA

**Keywords:** acquired subglottic stenosis, airway management, multidisciplinary approach, pregnancy-related complications, subglottic stenosis

## Abstract

Subglottic stenosis (SGS) is a narrowing of the airway that can be congenital or acquired, often resulting from prior intubation, infection, or autoimmune diseases. When SGS occurs during pregnancy, it presents unique challenges for anesthetic management. Symptoms commonly mimic those caused by pregnancy-related airway changes, making diagnosis and treatment more challenging. Additionally, ensuring both maternal and fetal safety during anesthesia is critical and requires a multidisciplinary approach.

We report a case of a 26-year-old pregnant woman at 12 weeks’ gestation who presented with severe hyperemesis gravidarum resulting in acute respiratory failure with suspected esophageal perforation, necessitating intubation. A Gastrografin study later ruled out perforation, and she was successfully extubated after two days without surgical intervention. One month later, she was readmitted with progressive dyspnea and wheezing, initially misdiagnosed as an asthma exacerbation. Her symptoms failed to improve with IV steroids, and a bedside fiberoptic exam by ENT revealed 85% SGS. Balloon dilation was performed under general anesthesia with a carefully tailored anesthetic plan, including the use of nebulized lidocaine, propofol, sevoflurane, dexmedetomidine, ketamine, and jet ventilation to maintain spontaneous ventilation and optimize maternal and fetal safety. Postoperatively, her respiratory symptoms improved, and she went on to have an uneventful delivery five months later.

This particular case highlights the critical role of anesthesia in managing SGS in pregnancy. Key considerations included maintaining stable oxygenation, preventing airway trauma, and ensuring minimal risk to the fetus. Balloon dilation proved effective in restoring airway patency. This case underscores the importance of a multidisciplinary approach to anesthesia care and emphasizes the need for individualized anesthetic strategies when managing airway conditions like SGS in pregnant patients.

## Introduction

Subglottic stenosis (SGS) is a narrowing of the airway below the vocal cords and above the trachea. It can be congenital, often linked to syndromes such as Down syndrome and CHARGE syndrome, or acquired due to prolonged intubation, trauma, autoimmune diseases (e.g., granulomatosis with polyangiitis and sarcoidosis), infections, or gastroesophageal reflux disease (GERD) [1]. Idiopathic SGS predominantly affects previously healthy, perimenopausal women, with an incidence of 1 per 400,000 annually [2]. SGS typically results from chronic inflammation and fibrosis, leading to progressive airway narrowing and, in severe cases, potential airway collapse [1]. SGS in pregnancy is rare and often misdiagnosed as asthma or recurrent bronchitis due to overlapping symptoms such as progressive dyspnea, stridor, hoarseness, and exertional oxygen desaturation [3]. Pregnancy-induced airway edema and increased oxygen demand can further exacerbate symptoms, complicating management. Diagnosis is made with endoscopic examination using flexible fiberoptic laryngoscopy [1]. Mild cases may respond to medical therapy, including corticosteroids, proton pump inhibitors for GERD, and immunosuppressive agents in autoimmune-related cases. However, symptomatic or severe stenosis often necessitates surgical intervention. Endoscopic approaches, such as balloon dilation, laser excision, and cold knife procedures, are commonly used to restore airway patency, while advanced cases may require open surgical procedures such as laryngotracheal reconstruction or tracheal resection with anastomosis [2]. Pregnancy adds further challenges, as airway management must balance maternal stability with fetal safety while minimizing teratogenic risks. No standardized guidelines exist for SGS in pregnancy, necessitating individualized, multidisciplinary care. This case highlights a successful approach to managing symptomatic SGS in a pregnant patient at 18 weeks’ gestation, emphasizing the importance of early recognition, interdisciplinary collaboration, and a tailored anesthetic strategy.

## Case presentation

A 26-year-old woman, G4P2012 at 18.1 weeks by nine-week ultrasound, presented with acute asthma exacerbation and progressive shortness of breath. The patient reported that the shortness of breath started approximately one month prior after being admitted at an outside hospital for management of hyperemesis gravidarum complicated by a syncopal episode. Imaging during that admission showed pneumomediastinum, prompting intubation given concern for esophageal perforation. After two days, she was extubated without surgical intervention following a Gastrografin study that ruled out perforation. Additional past medical history was significant for asthma, which had been well controlled with no exacerbations since childhood. Home medications included albuterol as needed, budesonide/formoterol twice a day, and prenatal multivitamins. 

**Figure 1:**
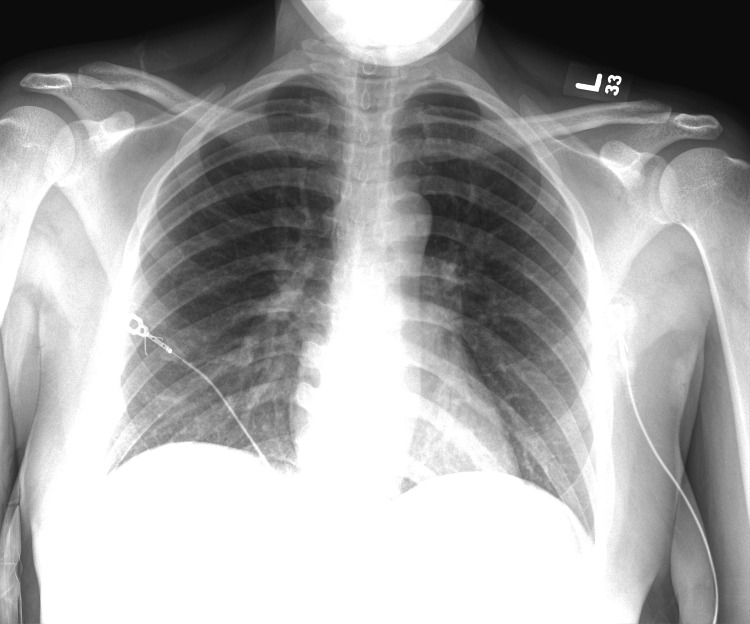
Normal chest radiograph at the initial presentation

At the time of presentation, she reported difficulty breathing, cough, and wheezing, and a physical exam revealed bilateral wheezes, stridor, neck muscle retraction, and tachypnea with a respiratory rate of 22 bpm. Her shortness of breath was worse when lying down and her oxygen saturation was 100% in room air and 92% with exertion. Chest X-ray revealed no acute abnormalities (Figure 1). What was thought to be an acute asthma exacerbation was treated with IV steroids, but there was no improvement. ENT consultation and a bedside fiberoptic exam revealed 85% SGS, with the scope unable to advance past the vocal cords. 

**Figure 2:**
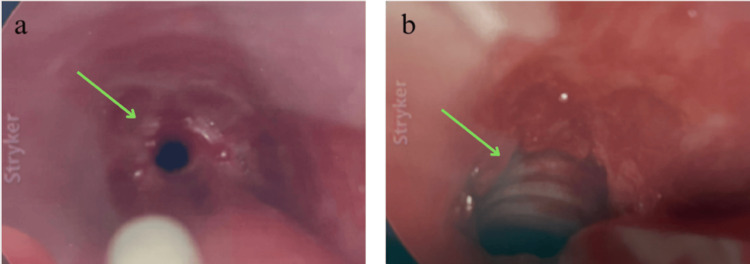
Rigid bronchoscopy images obtained near the site of subglottic stenosis a. Circumferential subglottic stenosis visualized before dilation, demonstrating narrowing of the airway lumen and limited airway patency. b. Magnified image depicts the airway following balloon dilation, highlighting improved lumen diameter and enhanced patency.

**Figure 3:**
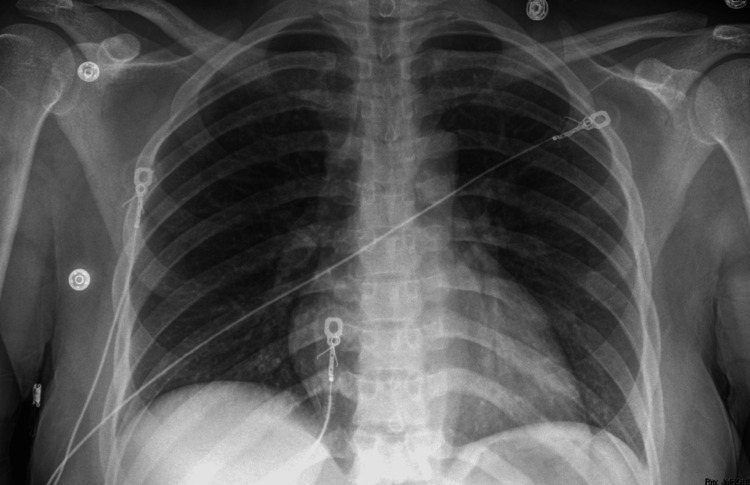
Normal chest radiograph following subglottic stenosis intervention

**Figure 4:**
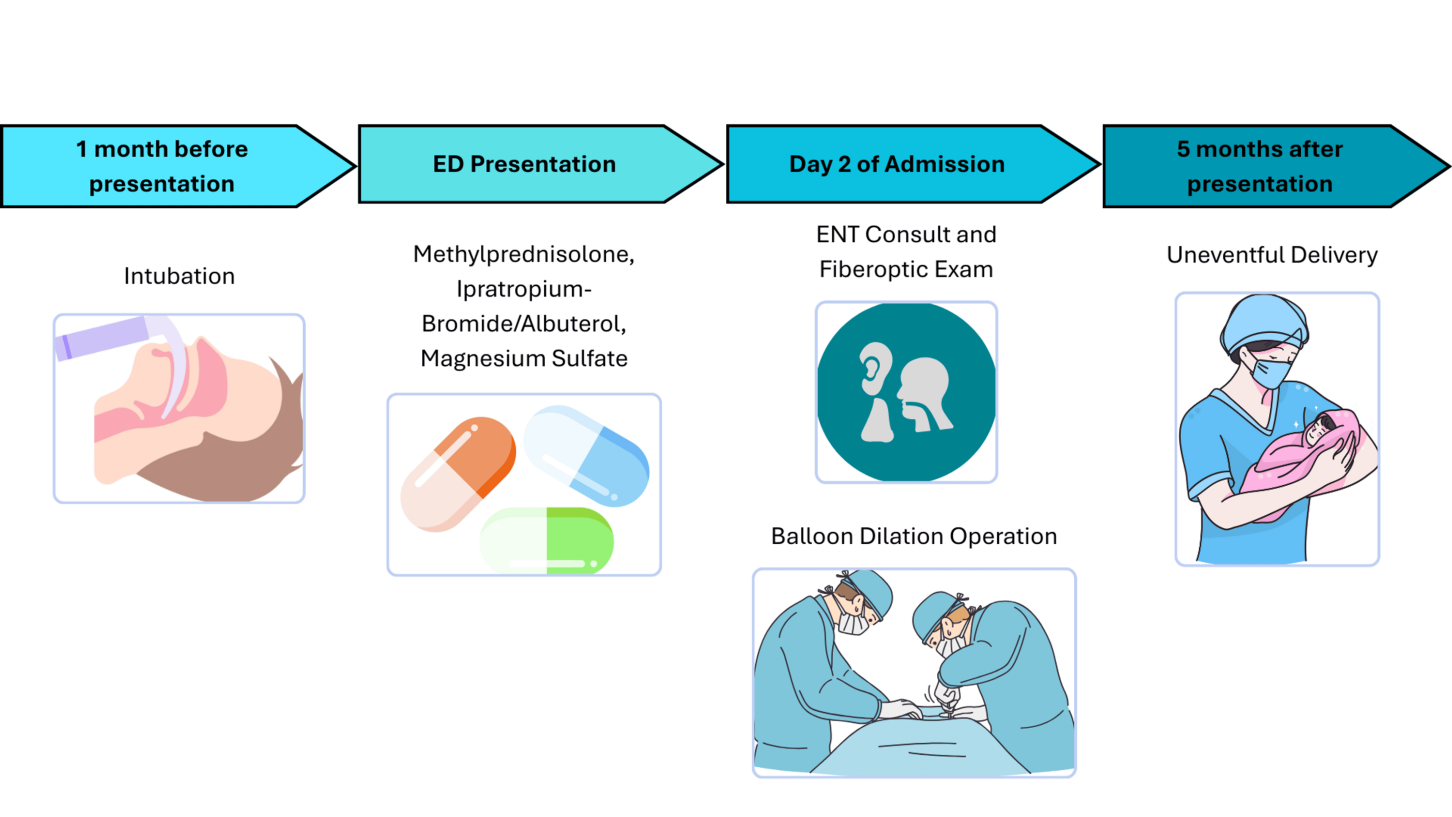
Sequence of diagnostic evaluation and therapeutic management Image Credits: Linh Ton

The patient was transferred to the ICU for monitoring and was scheduled for balloon dilation with ENT. Obstetrics was consulted for multidisciplinary care given the patient’s 18-week pregnancy. Intraoperative fetal monitoring was not used, after consultation with the obstetric service and review of The American College of Obstetricians and Gynecologists guidelines recommended continuous fetal heart rate monitoring starting at 23 to 24 weeks gestation. However, fetal heart tones were obtained prior to and after the procedure. Teratogenic medications were avoided. The airway was first topicalized with nebulized lidocaine and 1 mL of racemic epinephrine prior to induction. Propofol was titrated by increments of 20 mg to maintain spontaneous ventilation. Sevoflurane, dexmedetomidine, and ketamine were also titrated to maintain spontaneous ventilation. An endotracheal tube (ETT) could not be passed initially, so an 8 French Cook catheter (at FiO2 100%, PSI 30) was used in conjunction with a handheld ventilator to conduct jet ventilation. High magnification with a rigid telescope revealed approximately 70-75% stenosis in a circumferential pattern that began at 5 cm below the vocal cords and extended approximately 1 cm (Figure 2a). The ENT surgeon performed rigid bronchoscopy to balloon dilate the airway (Figure 2b). After successful dilation, the surgeon intubated the patient with a 6.0 ETT. Peripheral oxygen saturation levels were maintained at 92-100% throughout the procedure. The procedure lasted approximately one hour, during which the patient remained hemodynamically stable, with mean arterial pressures ranging from 65 to 92 mm Hg. No significant events were noted during anesthetic emergence and the ETT was subsequently removed by the anesthesia provider once the patient met extubation criteria. Postoperatively, the patient reported an improvement in her respiratory symptoms, with no further stridor or neck muscle retraction at the time of discharge, two days after the surgery. A post-procedure chest X-ray demonstrated normal lung fields and no acute abnormalities (Figure 3). She did not require readmission for respiratory distress and her delivery five months later was uneventful (Figure 4).

## Discussion

Acquired SGS in pregnancy is an uncommon and challenging clinical scenario, presenting with unique challenges due to concern for both maternal and fetal safety. The majority of acquired SGS cases are due to iatrogenic trauma, including prolonged intubation or injury during prior endotracheal intubation. Intubation can cause local microenvironmental changes leading to dysregulated tissue repair through mucosal inflammation, hypoxia, and biomechanical stress [4]. The hormonal and anatomical changes of pregnancy may also predispose pregnant women to airway edema and increased airway resistance. Specifically, increased blood volume and mucosal edema may exacerbate airway narrowing [5]. Differential expression of hormone receptors *ESR1*, *ESR2*, and *PGR* in the proximal airway mucosa of non-pregnant patients with idiopathic SGS has been noted, suggesting a possible hormonal influence [6]. 

Diagnosis of SGS in pregnancy can present certain challenges, given the overlapping clinical features with more common pregnancy-related conditions. Non-specific symptoms of progressive dyspnea, stridor, or hoarseness may be mistaken for pregnancy-related dyspnea or asthma exacerbations [7]. Cognitive biases, including anchoring, recency, and availability bias, may also contribute to potential misdiagnoses. Delays in recognition may then lead to shortcomings in timely diagnosis and treatment. Additional diagnostic challenges include an avoidance of typical imaging techniques, including CT scans and MRIs, given ongoing concerns of fetal radiation exposure. Difficulty with airway visualization during fiberoptic exam may also be encountered due to the anatomical changes in pregnancy, such as weight gain and pharyngeal edema [8]. 

Moreover, challenges during intraoperative management include ensuring effective airway management while simultaneously accommodating the surgeon’s need for airway access during the procedure. Determining the airway approach, surgical intervention, and obstetric considerations requires multidisciplinary planning, a key component to managing SGS in pregnancy effectively. Laryngeal dilation with continuous radial expansion balloons, paired with jet ventilation or high-flow nasal oxygenation, has been demonstrated as safe and effective in pregnant patients with SGS, supporting its role as a first-line option [9,10]. Endoscopic balloon dilation specifically has shown efficacy in restoring airway patency in pregnant patients while minimizing risk [11,12]. Alternative strategies described in the literature include tracheostomy and open surgical resection, although these carry higher morbidity and are typically reserved for refractory or recurrent cases [13]. Notably, one reported case of severe SGS was successfully treated with elective tracheostomy before administration of labor analgesia [13]. Surgical protocols vary by center and may include obstetrical consultation, preoperative and postoperative external fetal heart rate monitoring, continuous fetal heart rate monitoring, high-flow nasal cannula, and jet ventilation, tailored to balance airway access with maternal and fetal safety [9,14]. Further studies are needed to better define best practices for managing acquired SGS during pregnancy and to improve outcomes in this complex and rare clinical scenario.

## Conclusions

SGS in pregnancy is a rare and challenging condition that requires prompt recognition and specialized management to prevent life-threatening airway compromise. This case demonstrates that endoscopic balloon dilation, performed with a carefully tailored anesthetic plan, can safely and effectively relieve severe SGS during pregnancy without jeopardizing maternal or fetal safety. This successful outcome highlights the importance of early diagnosis, individualized anesthetic management, and a coordinated multidisciplinary approach involving ENT, anesthesia, and obstetrics teams. While this report represents a single case, it provides valuable clinical insights into a complex scenario. Additional studies are needed to establish evidence-based protocols for managing this rare condition in pregnancy.
